# Selenoneine Ameliorates Hepatocellular Injury and Hepatic Steatosis in a Mouse Model of NAFLD

**DOI:** 10.3390/nu12061898

**Published:** 2020-06-26

**Authors:** Masaaki Miyata, Koki Matsushita, Ryunosuke Shindo, Yutaro Shimokawa, Yoshimasa Sugiura, Michiaki Yamashita

**Affiliations:** Department of Food Science and Technology, National Research and Development Agency, Japan Fisheries Research and Education Agency, National Fisheries University, 2-7-1, Nagata-Honmachi, Shimonoseki 759-6595, Japan; koki.matsushita12@gmail.com (K.M.); r.shindy1007@gmail.com (R.S.); decoboco87@gmail.com (Y.S.); ysugiura@fish-u.ac.jp (Y.S.); mic@fish-u.ac.jp (M.Y.)

**Keywords:** selenoneine, farnesoid X receptor, non-alcoholic fatty liver disease, selenium, steatosis

## Abstract

Selenoneine is a novel organic selenium compound markedly found in the blood, muscles, and other tissues of fish. This study aimed to determine whether selenoneine attenuates hepatocellular injury and hepatic steatosis in a mouse model of non-alcoholic fatty liver disease (NAFLD). Mice lacking farnesoid X receptor (FXR) were used as a model for fatty liver disease, because they exhibited hepatomegaly, hepatic steatosis, and hepatic inflammation. *Fxr*-null mice were fed a 0.3 mg Se/kg selenoneine-containing diet for four months. Significant decreases in the levels of hepatomegaly, hepatic damage-associated diagnostic markers, hepatic triglycerides, and total bile acids were found in *Fxr*-null mice fed with a selenoneine-rich diet. Hepatic and blood clot total selenium concentrations were 1.7 and 1.9 times higher in the selenoneine group than in the control group. A marked accumulation of selenoneine was found in the liver and blood clot of the selenoneine group. The expression levels of oxidative stress-related genes (*heme oxygenase 1* (*Hmox1*), *glutathione S-transferase alpha 1* (*Gsta1*), and *Gsta2*), fatty acid synthetic genes (*stearoyl CoA desaturase 1*(*Scd1*) and *acetyl-CoA carboxylase 1* (*Acc1*)), and selenoprotein (*glutathione peroxidase 1* (*Gpx1*) and *selenoprotein P* (*Selenop*)) were significantly decreased in the selenoneine group. These results suggest that selenoneine attenuates hepatic steatosis and hepatocellular injury in an NAFLD mouse model.

## 1. Introduction

Selenium is an essential trace element with important metabolic functions in human health, including antioxidative and anti-inflammatory functions [1,2]. The selenium content in foods and dietary supplements exist in different chemical forms (organic and inorganic selenocompounds), such as selenomethionine, selenocysteine, selenite, selenious acid, and sodium selenite. Most studies on the health effects of selenium as nutrients were carried out using these organic and inorganic selenocompounds. The nutritional availability of selenium is highly dependent on its chemical form, because this affects absorption, distribution, metabolism, and excretion [3,4]. Furthermore, the health effects of selenium are dependent on the selenium species ingested [5,6].

**Figure 1:**
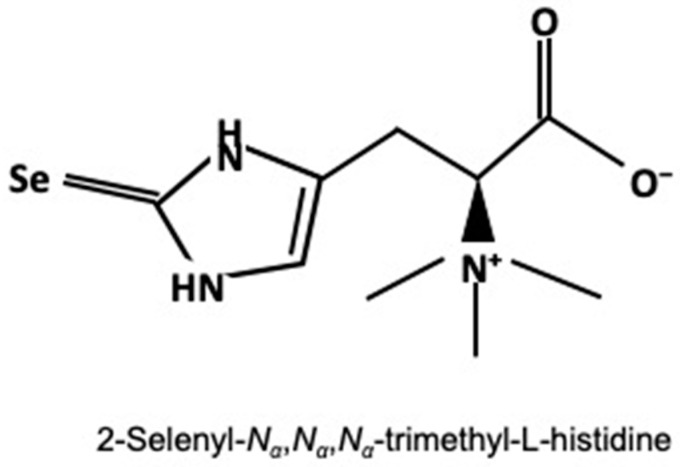
Chemical structure of selenoneine.

Selenoneine (2-selenyl-*N_α_,N_α_,N_α_*-trimethyl-l-histidine), an organoselenium compound, was isolated from the blood of bluefin tuna, *Thunnus orientalis* [7]. Selenoneine contains a selenium atom on the imidazole ring (Figure 1) and is a selenium analog of ergothioneine, which is a putative antioxidant compound acquired by animals through dietary sources [8,9]. It is widely distributed in various animal tissues and particularly exhibits at high levels in fish tissues, such as tuna, mackerel, and marlin [10]. Selenoneine was identified as the major organic organoselenium compound in the blood and muscle tissue of tuna [7,10]. Most organic selenium (98%) occurs as selenoneine in tuna muscle. Red tuna muscles contain selenoneine at 190 nmol Se/g, whereas tuna and mackerel blood contained it at more than 400 nmol Se/g [11]. Epidemiologic studies indicated that selenoneine is the major selenium species in the red blood cells of Canadian Inuit ingesting country food, such as fish and marine mammals [12]. Selenoneine has strong radical-scavenging activity in vitro. They measured the 50% radical-scavenging concentration with 1-diphenyl-2-picrylhydrazyl for the water-soluble vitamin E-like antioxidant Trolox, l-ergothioneine, and the reduced selenoneine form, and the results were 880, 1700, and 1.9 μM, respectively, indicating that selenoneine has a greater antioxidant activity than Trolox and 1-ergothioneine [10]. Selenoneine attenuates peroxide-induced oxidative stress in *C. elegans* [13] and methylmercury-mediated toxicity in zebrafish embryo and human cells [14]. Recently, dietary supplementation of selenoneine-containing tuna dark muscle extract has been shown to effectively reduce the pathology of experimental colorectal cancers in mice [15]. However, an evaluation of the beneficial health effect in vivo using purified selenoneine has not been performed to date.

In the present study, mice lacking farnesoid X receptor (FXR) were used to evaluate the beneficial health effect of purified selenoneine. FXR is a bile acid nuclear receptor, which plays an important role in lipid and glucose metabolism regulation [16,17]. Mice lacking FXR (*Fxr*-null mice) developed hepatic steatosis, chronic inflammation, and insulin resistance, and their livers showed elevated levels of triglycerides (TG), free fatty acids (FFA), bile acids, and total cholesterol (TC) [18,19,20,21]. Furthermore, hepatomegaly and elevated hepatic damage-associated diagnostic markers, such as serum alanine aminotransferase (ALT) and alkaline phosphatase (ALP), were observed in *Fxr*-null mice [18,22]. These parameters in *Fxr*-null mice were increased in an age-dependent manner [20]. Gene expression analyses have shown that the hepatic expression of pro-inflammatory cytokines and oxidative stress genes were upregulated in *Fxr*-null mice [20,21,23]. Hence, these mice were considered as models of non-alcoholic fatty liver disease (NAFLD). NAFLD is currently considered the most common liver disease and is characterized by excessive fat accumulation in the liver. It ranges from simple steatosis to a more aggressive form, non-alcoholic steatohepatitis, and may progress into hepatic fibrosis, cirrhosis, or hepatocellular carcinoma [24,25].

The researchers recently used *Fxr*-null mice for evaluating typical marine-derived ingredients, fish oil- and taurine-mediated preventive effects on NAFLD. Fish oil and taurine attenuated hepatocellular injury and hepatic steatosis in *Fxr*-null mice [26,27,28]. In the present study, selenoneine, an organic selenium compound, was used to evaluate whether selenocompound feeding ameliorates disrupted liver functions. *Fxr*-null mice were fed with a 0.3-mg Se/kg selenoneine-containing diet for four months. The results suggested that selenoneine feeding ameliorated hepatocellular injury and hepatic steatosis in the NAFLD mouse model.

## 2. Materials and Methods

### 2.1. Materials

*Fxr*-null mice were kindly provided by Dr. Frank J. Gonzalez (National Institute of Health, Bethesda, MD) [18]. Selenoneine was purified from the red muscle of yellowfin tuna as previously described [7].

### 2.2. Animal Treatment, Sample Collection, and Histological Analysis

The *Fxr*-null mice were housed under a standard 12 h light–dark cycle (7 a.m.–7 p.m.) Age-matched groups of 4-month-old male mice were used for all experiments. Eight mice for each group were used. The mice were fed a standard rodent chow (MF; Oriental Yeast Co. Ltd., Tokyo, Japan) originally containing 0.4 mg Se/kg selenium or 0.3 mg Se/kg selenoneine-containing MF diet for 4 months. Blood was taken via their tail every month. The mice were killed after 4 months of feeding. Liver tissues were fixed in 10% neutral buffered formalin and embedded in paraffin. Sections were prepared and stained with hematoxylin and eosin (H&E). All experiments were performed in accordance with the guidelines for animal experiments of the National Fisheries University (Shimonoseki, Japan). The protocol was approved by the Institutional Animal Care and Use Committee at the National Fisheries University (Permission No. 2018-18-2).

### 2.3. Determination of Selenoneine and Selenium Concentrations

The tissue samples (0.05 g) were weighed and mixed with 1.5 mL of 61% nitric acid (analytical grade; Fujifilm Wako Pure Chemical Co., Osaka, Japan) and 0.5 mL of 35% hydrogen peroxide (ultrapure grade, Fujifilm Wako Pure Chemical Co., Osaka, Japan) in a Diditube digestion tube. The mixtures were heated to 100 °C in 5 h. The solutions were diluted with ultrapure water. Selenium concentration was measured by inductively coupled plasma mass spectrometry (ICP-MS) (Agilent 7500; Agilent Technologies, Santa Clara, CA, USA). Selenium standard solution (1000 mg L^−1^, Fujifilm Wako Pure Chemical Co., Osaka, Japan) was used as selenium standard. Analysis was performed in duplicate for each sample. The selenoneine concentration was determined according to the method of Yamashita and Yamashita (2010). Chromatographic separation was carried out using a high-performance liquid chromatography (HPLC) pump (Agilent 1100, Agilent Technologies, Santa Clara, CA, USA). The analytical column was an MSpak GF-310 4D (4.6 mm × 150 mm, Showa Denko, Tokyo, Japan) equilibrated with 100-mM ammonium formate buffer containing 0.1% Igepal CA630. The injection volume was fixed at 10 μL. The mobile phase was isocratically delivered at 0.5 mL/min, and selenium was detected using online liquid chromatography inductively coupled plasma mass spectrometry (LC–ICP-MS; Agilent 7500) with a concentric nebulizer and a sample injector (2 mm id, quartz) monitoring 82Se, according to the method previously described [7]. The plasma and auxiliary argon gas flow rates were 0.7 and 0.3 L/min, respectively. The nebulization argon gas flow rate was 10.1 L/min. The radio frequency power was 1500 W. The selenoneine that was purified from bluefin tuna blood was used as a standard.

### 2.4. Determination of Hepatic Damage-Associated Diagnostic Markers and Hepatic Lipid Levels

Serum ALT and aspartate aminotransferase (AST) levels were determined using Transaminase CII-B-test Wako and ALP levels were Alkali-phospha B-test Wako commercial kits (Wako Pure Chemicals, Osaka, Japan). The hepatic samples were prepared as previously described [29]. Hepatic TG, FFA, and TC were determined using the Triglyceride E-test Wako, NEFA E-test Wako, and Cholesterol E-test Wako kits (Wako Pure Chemicals), respectively.

### 2.5. Determination of mRNA Levels

The hepatic total RNA was isolated by the acid guanidine–phenol–chloroform method. Single-strand cDNAs were synthesized using an oligo(dT) primer and the High-Capacity cDNA Reverse Transcription Kit (Applied Biosystems, Foster City, CA, USA). The cDNA was used for real-time quantitative polymerase chain reaction (qPCR) using SYBR Premix Ex Taq^TM^ II (Tli RNaseH Plus) (Takara Bio, Otsu, Japan) with the TP870 Thermal Cycler Dice Real Time System (Takara Bio). The relative mRNA levels were calculated by the comparative threshold cycle method and normalized to β-actin. The specific forward and reverse primers used in real-time qPCR are listed in Table S1.

### 2.6. Statistical Analysis

Data are presented as means ± SD. In animal experiments, the statistical significance was analyzed using Student’s *t*-test or a one-way ANOVA test followed by Dunnett’s test using the software Excel Statistics 2015 (Social Survey Research Information Co. Ltd., Tokyo, Japan). Differences with *p* values < 0.05 were considered statistically significant.

## 3. Results

### 3.1. Body and Liver Weights

**Figure 2:**
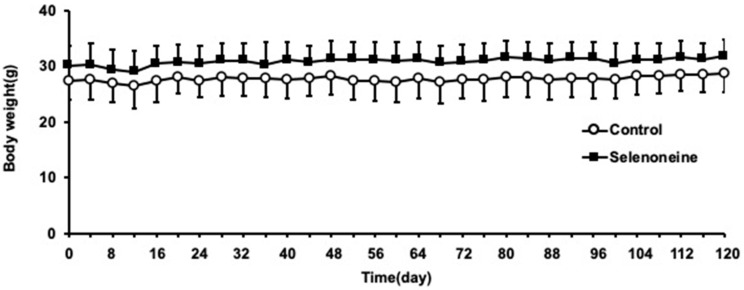
Changes in body weight. Mean body weight was shown at an interval of 4 days. Values are presented as mean ± SD (*n* = 8).

*Fxr*-null mice were fed with a standard rodent chow (MF) or a 0.3-mg Se/kg selenoneine-containing standard rodent chow for four months. The average body weight and food consumption in the selenoneine group were slightly higher than those in the control group during the feeding period (Figure 2). No significant alteration in food consumption and body weight were found between both groups during the feeding period. *Fxr*-null mice exhibited hepatomegaly, which resulted in increased liver weight. The ratio of liver weight to body weight of the selenoneine group was significantly lower than that of the control group, because the average liver weight in the selenoneine group was lower and the average body weight was higher than those in the control group (Table 1) [22].

### 3.2. Total Selenium and Selenoneine Levels

**Figure 3:**
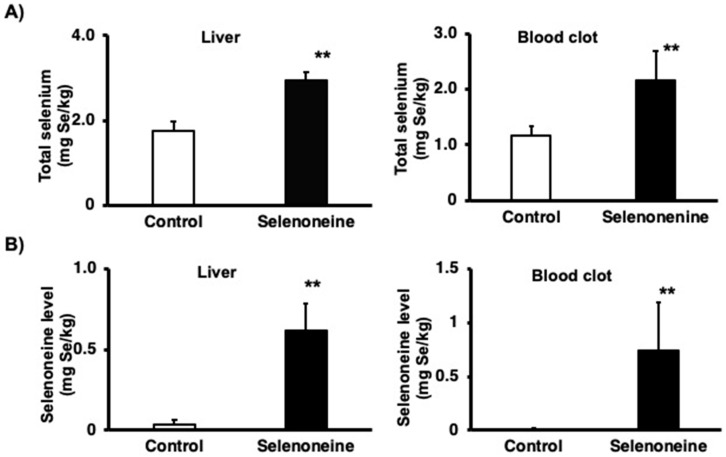
Total selenium and selenoneine concentration. (**A**) Total selenium level. (**B**) Selenoneine level. Mice were fed a diet supplemented with 0.3 mg Se/kg selenoneine for 4 months. The total selenium and selenoneine concentrations were measured using LC-ICP-MS. Values are presented as mean ± SD (*n* = 8). Significant differences were assessed using Student’s *t*-test (**, *p* < 0.01).

The hepatic and blood clot total selenium and selenoneine levels were measured with LC–ICP-MS to identify whether selenium and selenoneine accumulated in the liver and blood of mice fed with a selenoneine-containing diet for four months. The hepatic total selenium concentration was 1.7 times higher in the selenoneine group than in the control group (Figure 3A). Blood clot total selenium concentration was also 1.9 times higher in the selenoneine group than that in the control group. Selenoneine was detected (0.04 mg Se/kg liver) in the liver of control mice. The hepatic selenoneine concentration was more than 16 times higher in the selenoneine group than in the control group (Figure 3B). Selenoneine is known to accumulate in erythrocytes. Blood clot selenoneine concentration was less than 0.01 mg Se/kg in the control group, whereas it was 0.74 mg Se/kg wet cell in the selenoneine group.

### 3.3. Hepatic Damage-Associated Diagnostic Marker

**Figure 4:**
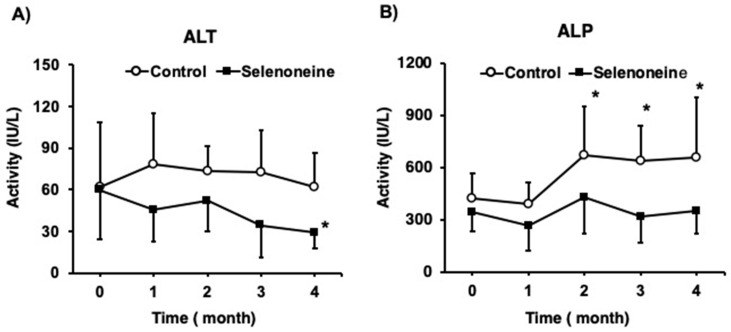
Changes in alanine aminotransferase (ALT) and alkaline phosphatase (ALP) activity. (**A**) ALT activity. (**B**) ALP activity. Mice were supplemented with 0.3-mg Se/kg selenoneine for 4 months. Blood was taken from mouse tail every month. Values are presented as mean ± SD (*n* = 8). Significant differences were assessed Dunnett’s test (* *p* < 0.05 vs. corresponding 0 month mice).

**Figure 5:**
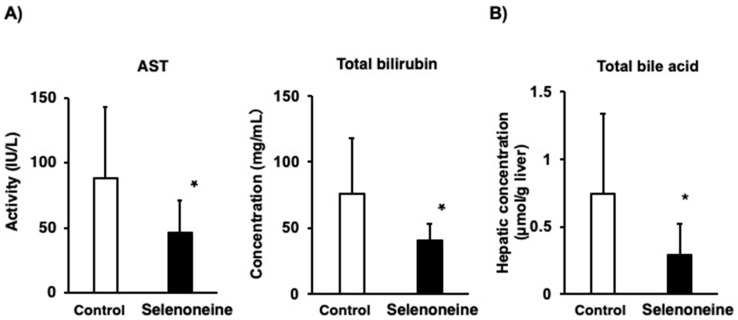
Changes in hepatic damage-associated diagnostic markers. (**A**) Aspartate aminotransferase (AST) and total bilirubin. (**B**) Total bile acid. Mice were supplemented with 0.3-mg Se/kg selenoneine for 4 months. Values are presented as mean ± SD (*n* = 8). Significant differences were assessed using Student’s t-test (*, *p* < 0.05 vs. corresponding control mice).

Elevated hepatic damage-associated diagnostic markers, such as serum ALT and ALP activities, were found in *Fxr*-null mice [22]. Time-course analyses of hepatic damage-associated diagnostic markers were performed. ALT activity was not altered in the control group during the four-month period, but it was significantly decreased in the selenoneine group at four months (Figure 4A). ALP activity was significantly increased in the control group at 2, 3, and 4 months, whereas it was not altered in the selenoneine group during the four-month period (Figure 4B). AST activity and total bilirubin concentration were also significantly lower in the selenoneine group than in the control group at four months (Figure 5). *Fxr*-null mouse livers showed elevated levels of total bile acids due to the lack of FXR signaling. The hepatic total bile acid concentration was significantly lower in the selenoneine group than in the control group.

### 3.4. Hepatic and Serum Lipid Levels

**Figure 6:**
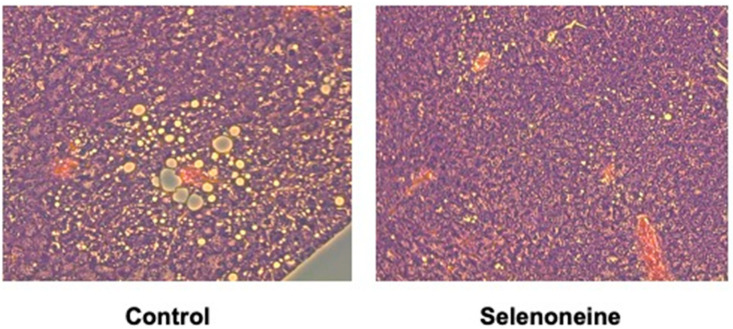
Influence of selenoneine feeding on hepatic histology in *Fxr*-null mice. Mice were supplemented with 0.3-mg Se/kg selenoneine for 4 months. Representative H&E-stained livers of *Fxr*-null mice were shown. Original magnification: 100 X.

**Figure 7:**
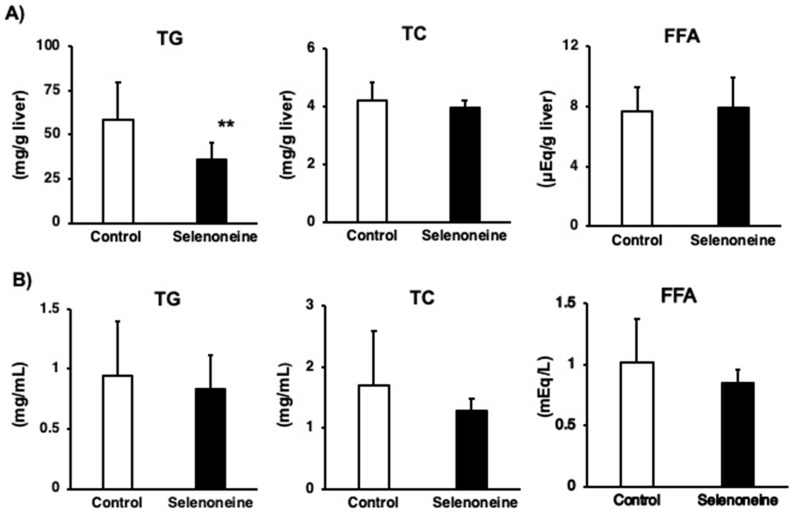
Changes in hepatic and serum lipid levels. (**A**) Hepatic lipid levels. (**B**) Serum lipid levels. Mice were supplemented with 0.3-mg Se/kg selenoneine for 4 months. Values are presented as mean ± SD (*n* = 8). Significant differences were assessed by the Student’s *t*-test (**, *p* < 0.01 vs. corresponding control mice). TG, triglyceride; TC, total cholesterol; FFA, free fatty acid.

**Figure 8:**
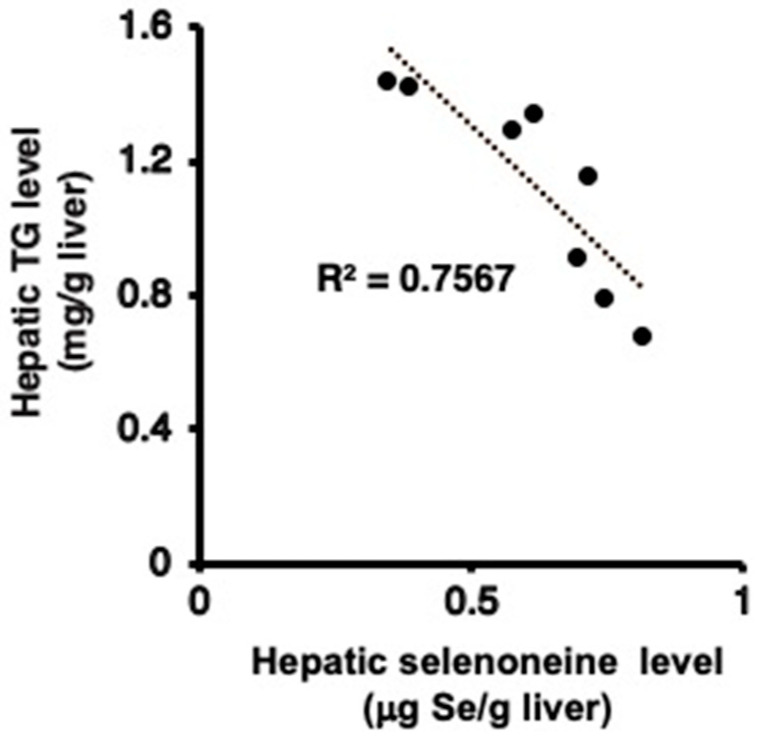
Correlation of hepatic selenoneine concentration with hepatic TG levels. Mice were supplemented with 0.3-mg Se/kg selenoneine for 4 months.

*Fxr*-null mice developed hepatic steatosis, and their livers and serum showed elevated levels of TG, FFA, and TC [18,21,27]. Significant alterations in hepatic morphology were not found in hematoxylin and eosin (H&E) staining of liver sections between the control and selenoneine group (Figure 6). However, H&E staining of liver sections showed several vacuoles due to lipid depositions in the control group but not in the selenoneine group, supporting that selenoneine feeding ameliorates hepatic steatosis. Furthermore, hepatic and serum lipid levels were measured to identify whether hepatic steatosis and dyslipidemia were reduced in the selenoneine group. Consistent with the results of H&E staining, hepatic TG level was significantly decreased in the selenoneine group, whereas hepatic TC and FFA levels were not significantly changed in the selenoneine group (Figure 7A). Serum TG, TC, and FFA levels were not also decreased in the selenoneine group (Figure 7B). Correlation analyses were performed to examine whether hepatic selenoneine accumulation is related to reduced hepatic TG levels. There were significant inverse correlations (R^2^ = 0.686) between hepatic selenoneine and hepatic TG levels (Figure 8).

### 3.5. Hepatic Gene Expression Levels

To explore the crucial mechanisms involved in the selenoneine-mediated reversion to hepatocellular injury and hepatic steatosis, changes in the mRNA levels of pro-inflammatory cytokine genes (*tumor necrosis factor α* (*Tnfα*), *interleukin-1β* (*Il-1β*), and *interleukin-6* (*Il-6*)) and oxidative stress-related genes (*heme oxygenase 1* (*Hmox1*), *glutathione S-transferase alpha 1* (*Gsta1*), *and glutathione S-transferase alpha 2* (*Gsta2*)) were analyzed (Table 2). Selenoneine feeding significantly decreased the mRNA levels of three oxidative stress-related genes (*Hmox1*, *Gsta1*, and *Gsta2*) but not those of the pro-inflammatory cytokine genes (*Tnfα*, *Il-1β*, and *Il-6*) in *Fxr*-null mice. To explore the mechanisms involved in the selenoneine-mediated improvement of disrupted hepatic lipid metabolism in *Fxr*-null mice, changes in the mRNA levels of lipogenic (*fatty acid synthase* (*Fasn*), *stearoyl CoA desaturase 1* (*Scd1*), and *acetyl-CoA carboxylase 1* (*Acc1*)), bile acid metabolizing (*bile salt export pump* (*Bsep*) and *cholesterol 7α-hydroxylase* (*Cyp7a1*)), and lipid metabolism-regulating (*sterol regulatory element-binding protein 1c* (*Srebp1c*) and *peroxisome proliferator-activated receptor alpha* (*Pparα*)) genes were analyzed (Table 3). Selenoneine feeding significantly decreased the mRNA levels of lipogenic genes (*Fasn*, *Scd1*, and *Acc1*). The level of *Srebp1c* mRNA tended to decrease in the selenoneine group. Hepatic mRNA levels of selenium-containing protein (*selenoprotein P* (*Selenop*), *glutathione peroxidase 1* (*Gpx1*), *Gpx2*, *Gpx4*, and *thioredoxin reductase 1* (*Txnrd1*)) were analyzed, because hepatic selenium levels were significantly increased in the selenoneine group. Selenoneine feeding did not increase these mRNA levels. Conversely, it significantly decreased the mRNA levels of *Gpx1* and *Selenop*.

## 4. Discussion

This study demonstrated that selenoneine, an organic selenium compound remarkably found in the blood and muscle of fishes, ameliorated hepatocellular injury and hepatic steatosis in *Fxr*-null mice. Increased hepatic Nrf2 protein and hepatic *Hmox 1*, *Gsta1*, and *Gsta2* mRNA levels were found in *Fxr*-null mice, indicating that oxidative stress is spontaneously enhanced in *Fxr*-null mice [23]. The decreased mRNA levels of *Hmox 1*, *Gsta1*, and *Gsta2* suggest that selenoneine-mediated antioxidant activity is involved in the attenuation of hepatic dysfunction, because selenoneine has great antioxidant activity in vitro. On another note, selenoneine reversed a continuously high level of hepatic bile acids in *Fxr*-null mice. The decreased hepatic bile acid levels possibly led to reduced hepatic oxidative stress levels. The decreased serum ALP activity and bilirubin concentration indicated that selenoneine suppressed the progression of cholestatic liver disease in *Fxr*-null mice. Selenoneine’s biological effect, that is distinct from its antioxidative properties, may improve disrupted bile acid metabolism through the functional compensation of FXR signaling. Selenoneine feeding reversed elevated hepatic TG levels in *Fxr*-null mice. Furthermore, hepatic selenoneine concentration was inversely correlated with hepatic TG levels. Hepatic accumulation of selenoneine is partly involved in the amelioration of hepatic steatosis through reducing hepatic lipogenesis due to Acc1 and Scd1 expression. Several reports indicated that high-selenium diets enhanced the mRNA expression of Srebp1c, resulting in enhanced hepatic lipogenesis [30]. An updated epidemiologic study indicated that increased plasma selenium level is associated with the elevated prevalence of NAFLD [31]. The effect of selenoneine on NAFLD may differ from that of other selenium species.

The liver is the central organ for selenium regulation. It regulates whole-body selenium by producing excretory selenium forms and distributes selenium to other tissues by secreting selenoprotein P into the plasma [32]. Selenium compounds such as selenious acid and sodium selenite are known to be incorporated into several selenium-containing proteins (selenoproteins), which have important biological functions. These selenium intakes elevate activity or production of selenoproteins, such as *Gpx1* and *Selenop*, whereas selenium deficiency decreases these expressions. In the present study, however, increased hepatic mRNA levels of selenoproteins were not found in selenoneine feeding. Moreover, selenoprotein, *Gpx1*, *Gpx2*, *Gpx4*, *Txnrd1*, and *Selenop* mRNA levels were not increased, and *Gpx1* and *Selenop* mRNA levels were rather significantly decreased in the liver of mice supplemented with selenoneine under the condition of the present study. These results indicate that selenoneine feeding does not necessarily induce hepatic selenoprotein expression and, in some genes, decreases it. Furthermore, the selenoneine that was remarkably accumulated in the liver and blood of *Fxr*-null mice fed with selenoneine suggests that a large proportion of selenoneine remains unchanged in the liver and blood. These ideas are supported by the report that selenoneine is a major selenium species in the red blood cells of Inuit and Japanese people who frequently ingest marine food [12,33,34]. Hepatic selenoneine accumulation suggests that the attenuation of hepatocellular injury and hepatic steatosis is mainly due to hepatic selenoneine-mediated functions rather than other induced selenium-containing components, such as selenoproteins. Inorganic selenium, selenite, and selenate are incorporated into selenoproteins, resulting in elevated selenoproteins that exert several biological functions [32]. Other dietary forms of selenium, such as selenomethionine and selenocysteine, are also effectively incorporated into selenoproteins. Several reports suggest the favorable effect of selenium supplements, such as selenomethionine and sodium selenite, on hepatic dysfunction and hepatic steatosis [35,36]. Significant increases in the activity or mRNA levels of selenoproteins, such as *Gpx* and *Txnrd*, were found in these reports. Selenium acts indirectly as an antioxidant through its incorporation into selenoproteins, such as *Gpx1* and *Gpx4*. On another note, selenoneine seems to be hardly metabolized and incorporated into selenoproteins. The biological functions of selenium are likely dependent on its chemical form, and the functional properties of selenoneine may be different from those of other dietary selenium-containing components, such as selenomethionine, selenocysteine, selenite, and selenate.

Red muscle in tuna, mackerel, and marlin contains more than 5 mg Se/kg of selenoneine [7,11]. In the present study, mice were fed with a diet containing 0.3-mg Se/kg selenoneine. The low concentration of selenoneine supplementation attenuated hepatocellular injury and hepatic steatosis to a similar extent as 2% fish oil replacement on *Fxr*-null mice [26,28]. Fish oil components, such as docosahexaenoic acid (DHA) and eicosapentaenoic acid (EPA), are recognized primary materials highlighting the beneficial health effects of fish intake. The present study considers the possibility that selenoneine may also play an important role in health improvement due to its similar effects to beneficial compounds, such as DHA and EPA, which are mainly derived through fish intake. Epidemiologic studies suggest that blood and dietary selenium are reversely associated with the prevalence of stroke in Canadian Inuit who have a high selenium intake from the consumption of country food, such as fish and marine mammals [37]. Selenoneine is a major selenium species in the red blood cells of Canadian Inuit [12]. DHA and EPA also have beneficial effects in the treatment of NAFLD, including decreased hepatic mRNA levels of lipogenic genes [38,39]. Several functional similarities were found among DHA, EPA and selenoneine, selenoneine, and n-3 polyunsaturated fatty acids (n-3 PUFA), suggesting that they may be synergistically involved in attenuating NAFLD through regular fish intake.

Selenoneine feeding decreased hepatic *Gpx1* and *Selenop* mRNA levels, whereas excessive selenium consumption promoted the production of Gpx1 and Selenop, resulting in enhanced insulin resistance [40,41,42]. Recent studies indicate that excess *Gpx1* and *Selenop* exacerbate glucose metabolism and promote type 2 diabetes mellitus [43,44]. Increased *Gpx1* and *Selenop* are significant therapeutic targets for type 2 diabetes mellitus. Selenoneine significantly decreased hepatic *Gpx1* and *Selenop* mRNA levels in *Fxr*-null mice, which exhibit higher serum glucose and insulin resistance [19]. In a population with a high fish and seafood intake, fish consumption was associated with a lower risk of type 2 diabetes mellitus in men [45]. Identifying the influence of selenoneine intake on type 2 diabetes mellitus may be an important topic for future studies.

## 5. Conclusions

The researchers demonstrated that selenoneine ameliorates hepatocellular injury and hepatic steatosis in a mouse model of NAFLD. To the best of our knowledge, this is the first demonstration that purified selenoneine supplementation has beneficial health effects in mammalian models. Selenoneine, as well as n-3 PUFA, may play an important role in preventing NAFLD through regular fish intake. Further studies are necessary to explore the mechanism of the selenoneine-mediated prevention of NAFLD.

## Figures and Tables

**Table 1:** Body and hepatic weight.

Parameters	Control	Selenoneine
Body weight (g)	28.7 ± 3.2	31.8 ± 3.0
Liver weight (g)	2.15 ± 0.51	1.98 ± 0.29
Liver/body weight ratio (%)	7.49 ± 1.98	5.19 ± 0.59 *

Values are presented as mean ± SD (*n* = 8). Significant differences were assessed by the Student’s *t* test (*, *p* < 0.05).

**Table 2:** Hepatic mRNA levels of disorder-related genes.

Gene Symbol	Control	Selenoneine
*Tnf α*	1.00 ± 0.41	0.74 ± 0.66
*IL-1 β*	1.00 ± 0.65	0.61 ± 0.50
*IL-6*	1.00 ± 1.08	0.62 ± 0.55
*Hmox1*	1.00 ± 1.04	0.19 ± 0.23 *
*Gsta1*	1.00 ± 1.06	0.02 ± 0.03 *
*Gsta2*	1.00 ± 0.87	0.08 ± 0.08 *

Tnfα, tumor necrosis factor α; Il-1β, interleukin-1β; Il-6, interleukin-6; Hmox1, heme oxygenase 1; Gsta1, glutathione S-transferase alpha 1; Gsta2, glutathione S-transferase alpha 2. Values are presented as mean ± SD (*n* = 8). Significant differences were assessed by the Student’s *t*-test (*, *p* < 0.05).

**Table 3:** Hepatic mRNA levels of selenoprotein and lipid-related genes.

Gene Symbol	Control	Selenoneine
*Acc1*	1.00 ± 0.60	0.53 ± 0.42 *
*Scd1*	1.00 ± 0.55	0.24 ± 0.22 **
*Fasn*	1.00 ± 0.86	0.34 ± 0.26 *
*Ppar α*	1.00 ± 0.77	0.79 ± 0.54
*Srebp1c*	1.00 ± 0.67	0.47 ± 0.54
*Bsep*	1.00 ± 0.71	0.47 ± 0.34
*Cyp7a1*	1.00 ± 0.60	1.77 ± 1.47
*Gpx1*	1.00 ± 0.86	0.34 ± 0.35 *
*Gpx2*	1.00 ± 1.97	1.01 ± 1.65
*Gpx4*	1.00 ± 0.66	0.46 ± 0.50
*Txnrd1*	1.00 ± 0.78	0.49 ± 0.49
*Selenop*	1.00 ± 0.86	0.38 ± 0.34 *

Scd1, stearoy-CoA desaturase-1; Acc1, acetyle-CoA carboxylase-1; Fasn, fatty acid synthase; Pparα, peroxisome proliferator-activated receptor; Srebp1c, sterol regulatory element binding protein 1c; CYP7α1, cholesterol 7α-hydroxylase; Bsep, bile salt export pump; Gpx1, glutathione peroxidase 1; Selenop: selenoprotein P, Txnrd1: thioredoxin reductase 1. Values are presented as mean ± SD (*n* = 8). Significant differences were assessed by the Student’s *t*-test (*, *p* < 0.05; **, *p* < 0.01).

## Supplementary Materials

The following are available online at https://www.mdpi.com/2072-6643/12/6/1898/s1, Table S1: Primer sequences for real time PCR.
