# Supplementary material for: Selenoneine Ameliorates Hepatocellular Injury and Hepatic Steatosis in a Mouse Model of NAFLD
Source: Nutrients. 2020 Jun 26;12(6):1898. doi: 10.3390/nu12061898 (PMC7353312; doi:10.3390/nu12061898)
Supplement: Supplementary file 1 [file nutrients-12-01898-s001.pdf]

Supplementary Materials

Table S1. Primer sequences for real time PCR.

Gene	Sequence (forward)	Sequence (reverse)
Acc1	ACAGTGAAGGCTTACGTCTG	TGTGGACGATGGAGTCCATG
Bsep	TGGGCAGAAGCAAAGGGTAG	GCAGGATCTTGGGTTTCCGT
Cyp7a1	AGCAACTAAACAACCTGCCAGTACTA	GTCCGGATATTCAAGGATGCA
Fasn	CGGTCTGGAAAGCTGAAGGATC	CGGAGTGAGGCTGGGTTGA
Gpx1	CAGGAGAATGGCAAGAATGA	GAAGGTAAAGAGCGGGTGAG
Gpx2	ATCAAACGGCTCCTCAAAGT	GGGACGATATTCAGGGAATG
Gpx4	GCAGGAGCCAGGAAGTAATC	GGCTGGACTTTCATCCATTT
Gsta1	CAGCCTGGCAGCCAGAGA	TCTGTGGCTCCATCAATGCA
Gsta2	ACATGAAGGAGAGAGCCCTGAT	GCAGTCTTGGCTTCTCTTTGGT
Hmox1	CCTCACTGGCAGGAAATCATC	CCTGGTGGAGACGCTTTACATA
IL-1β	GCACTACAGGCTCCGAGATGAAC	TTGTCGTTGCTTGGTTCTCCTTGT
IL-6	CCACTCACCTCTTCAGAA	GCGCAAAATGAGATGAGT
Pparα	CCTCAGGGTACCACTACGGAGT	GCCGAATAGTTCGGCCGAA
Scd1	AGTTAACCACTGAATGCGAGG	GAGGTCCATTAGCACTTGCC
Selenop	CCTTGGTTTGCCTTACTCCTTCC	TTTGTTGTGGTGTTTGTGGTGG
Srebp1c	GGAGCCATGGATTGCACATT	GAAGTCACTGTCTTGGTTGTTG
Tnfα	TGTCTACTGAACTTCGGGGTGATC	GGTTGTCTTTGAGATCCATGCCGT
Txnrd1	AGGAACTCTGTCAGGACAGCCAGTA	GCCAGCATGTTCACGGTCA
β-actin	GCCAACACAGTGCTGTCTG	CCTGCTTGCTGATCCACATC
